# Cigarette Smoke Exposure Inhibits Bacterial Killing via TFEB-Mediated Autophagy Impairment and Resulting Phagocytosis Defect

**DOI:** 10.1155/2017/3028082

**Published:** 2017-12-28

**Authors:** Garrett Pehote, Manish Bodas, Kathryn Brucia, Neeraj Vij

**Affiliations:** ^1^College of Medicine, Central Michigan University, Mount Pleasant, MI, USA; ^2^Department of Pediatrics and Pulmonary Medicine, The Johns Hopkins University School of Medicine, Baltimore, MD, USA

## Abstract

**Introduction:**

Cigarette smoke (CS) exposure is the leading risk factor for COPD-emphysema pathogenesis. A common characteristic of COPD is impaired phagocytosis that causes frequent exacerbations in patients leading to increased morbidity. However, the underlying mechanism is unclear. Hence, we investigated if CS exposure causes autophagy impairment as a mechanism for diminished bacterial clearance via phagocytosis by utilizing murine macrophages (RAW264.7 cells) and *Pseudomonas aeruginosa* (PA01-GFP) as an experimental model.

**Methods:**

Briefly, RAW cells were treated with cigarette smoke extract (CSE), chloroquine (autophagy inhibitor), TFEB-shRNA, CFTR(inh)-172, and/or fisetin prior to bacterial infection for functional analysis.

**Results:**

Bacterial clearance of PA01-GFP was significantly impaired while its survival was promoted by CSE (*p* < 0.01), autophagy inhibition (*p* < 0.05; *p* < 0.01), TFEB knockdown (*p* < 0.01; *p* < 0.001), and inhibition of CFTR function (*p* < 0.001; *p* < 0.01) in comparison to the control group(s) that was significantly recovered by autophagy-inducing antioxidant drug, fisetin, treatment (*p* < 0.05; *p* < 0.01; and *p* < 0.001). Moreover, investigations into other pharmacological properties of fisetin show that it has significant mucolytic and bactericidal activities (*p* < 0.01; *p* < 0.001), which warrants further investigation.

**Conclusions:**

Our data suggests that CS-mediated autophagy impairment as a critical mechanism involved in the resulting phagocytic defect, as well as the therapeutic potential of autophagy-inducing drugs in restoring is CS-impaired phagocytosis.

## 1. Introduction

Chronic obstructive pulmonary disease (COPD) is characterized by chronic inflammation, emphysema, and recurring chronic infections of the lower airways [1–4]. Currently, COPD is the fourth leading cause of death in the United States and is expected to become the third largest cause globally by 2020 [1–3, 5]. One plausible reason for this increase in mortality is an increased prevalence of cigarette smoking, which is one of the major risk factors for COPD pathogenesis [1, 4].

As mentioned above, chronic infection is a major contributor to the worsening and progression of the obstructive lung disease. Specifically, the lower respiratory tract of COPD patients is often faced with bacterial colonization and viral infections. The most common bacterial pathogens responsible for these infections are *Streptococcus pneumoniae*, nontypeable *Haemophilus influenzae*, and *Pseudomonas aeruginosa* [2, 3, 6–9]. The presence of these pathogens and the resulting infections result in exacerbations that increase inflammation and decrease lung function, leading to the progression of COPD and resulting increase in hospitalization and mortality [2, 3, 6, 7, 10, 11]. Although the exact cause of chronic infections in COPD is unknown, there is increasing concentration on designing novel therapeutics to improve the morbidity of COPD subjects because chronic antibiotic treatments over time develop resistance [2, 7, 12–15].

A critical aspect of host defense against bacterial infections are highly phagocytic cells called macrophages [3, 7, 10, 16]. The ability of these cells to remove pathogens through phagocytosis is necessary for controlling debilitating lung infections [3, 7, 11, 16]. Therefore, phagocytosis is a necessary cellular process that recognizes foreign pathogens/particles and removes them [3, 7]. Our preliminary studies suggested that the chronic infections of the lower airway in COPD might involve dysfunction of the phagocytic ability of alveolar macrophages [2, 3, 10]. One possible mechanism for the diminished phagocytosis in alveolar macrophages is cigarette smoke- (CS-) induced autophagy impairment [2, 12–14, 17–19]. Briefly, autophagy is a homeostatic cellular process that degrades misfolded proteins, damaged organelles, and pathogens, which can be impaired by chronic CS exposure, the leading cause of COPD-emphysema pathogenesis [1, 12, 14–16, 19–21]. Despite studies that have demonstrated CS exposure may impair phagocytosis in macrophages [2, 9, 11], the exact mechanism remains unknown. Thus, in this study, we aimed to investigate the specific mechanism by which CS impairs bacterial clearance. We first focused on evaluating the role of transcription factor EB (TFEB), the master autophagy regulator that induces the transcription of various autophagy/lysosomal biogenesis genes based on our recent data suggesting its critical role in COPD-emphysema pathogenesis [15, 19, 22].

Briefly, these preliminary studies revealed that CS exposure induces localization of TFEB to aggresome bodies, which was associated with a decrease in lung function and increased severity of emphysema in COPD subjects. [15]. This finding led us to evaluate possible therapeutic approaches to induce TFEB expression as a way of inducing autophagy [15]. One drug that we have investigated for TFEB induction is a flavonoid called fisetin. Fisetin is an over-the-counter dietary supplement that acts as an antioxidant for brain health [15, 22, 23]. In recent studies from our lab and others, fisetin has been shown to induce TFEB and consequentially autophagy [15, 18, 19].

Hence, this study investigated further the mechanism of CS-impaired bacterial phagocytosis in COPD in order to explain the mechanism and reason for recurring exacerbations. First, we verified the pathogenic role of CS-induced autophagy impairment as a mechanism for diminished phagocytosis that may account for the chronic exacerbations in COPD. We also found that fisetin was effective in restoring CS-impaired bacterial phagocytosis. Moreover, fisetin also demonstrated an added therapeutic potential as a possible mucolytic and bactericidal.

## 2. Materials and Methods

### 2.1. Reagents and Treatments

The murine macrophage cell line, RAW264.7 was used as an *in vitro* model to investigate cigarette smoke exposure and its impact on phagocytosis. Standard cell culture procedures as previously described were used [14]. Briefly, cells were maintained at 37°C in 5% CO_2_ in DMEM/F12 media with 10% fetal growth serum (RMBIO) and 1% PS (penicillin and streptomycin; Invitrogen). For *in vitro* cigarette smoke exposure, cigarette smoke extract (CSE) was prepared by burning two to three 3R4F research-grade cigarettes (Tobacco Research Institute, University of Kentucky, Lexington, KY) and aerating into serum-free DMEM/F12 media (20 ml). An OD (320 nm) of 0.74 was considered to be 100% CSE, and working CSE concentrations were adjusted using cell culture media. As a model of CS-induced autophagy inhibition in RAW264.7 cells, chloroquine (30 *μ*M) was used for 8 hrs of pretreatments. To investigate the role of TFEB in the regulation of autophagy- and xenophagy-mediated phagocytosis, TFEB expression was knocked down in RAW cells by transfecting them with TFEB-Mission™ shRNA (Sigma) for 24 hrs using the Lipofectamine 2000 transfection reagent (Invitrogen, Carlsbad CA) following the protocol provided by the manufacturer. Similarly, cystic fibrosis transmembrane conductance regulator (CFTR) was inhibited in the macrophages using CFTR(inh)-172 inhibitor (Sigma-Aldrich, St. Louis, MO) at a concentration of 10 *μ*M for 8 hrs to examine the possible role of CFTR in CS-impaired phagocytosis.

#### 2.1.1. *Pseudomonas aeruginosa* Infection Model

To investigate if the phagocytic defect could be ameliorated by TFEB-mediated autophagy induction, fisetin treatment was utilized. Autophagy inducer, cysteamine (250 *μ*M; Sigma) was used as a positive control in the bactericidal and mucolytic experiments. For experiments involving infection, *Pseudomonas aeruginosa* (*P. aeruginosa*) strain *PA01*-GFP was cultured for 15–18 hrs in Luria Bertani (LB; Thermo Fisher Scientific, Waltham, MA) broth with carbenicillin (1%, Sigma) to select for PA01-GFP at 37°C and 250 rpm, in a shaking incubator. This culture of *PA01-*GFP was added directly to DMEM/F12 media in each well at a multiplicity of infection (MOI) of 10.

### 2.2. Immunoblotting

Our previously described immunoblotting method [20, 24] was used to quantify changes in the expression of TFEB (master autophagy regulator), CFTR, p62 (aggresome marker), and *β*-actin in the soluble protein fractions of RAW264.7 cells. TFEB was procured from Santa Cruz Biotechnology, CFTR-181 in-house [25] and *β*-actin from Sigma.

### 2.3. Fluorescence Microscopy for Quantification of Phagocytosis

RAW264.7 cells were plated onto 12 or 24 well plates and pretreated for 8 hrs with fisetin (20 *μ*M), CSE (5%), chloroquine (10 *μ*M), and CFTR(inh)-172 (inhibitor; 10 *μ*M) or transfected for 24 hrs with TFEB-Mission™ shRNA. After treatment, these cells were infected with *PA01-*GFP at an MOI of 10 for 3 hrs prior to fluorescence microscopy. Fluorescence images were captured using the ZOE™ Florescent Cell Imager (Bio-Rad). *PA*01-GFP-infected (fluorescent) and total (bright-field) macrophages in the same field were counted in order to analyze the phagocytic ability of the cells. The percentage of macrophages infected with *PA01*-GFP-infected macrophages as compared to the total number of macrophages was quantified to calculate the bacterial clearance by macrophages.

### 2.4. Bacterial Survival Assay

RAW cells were seeded into 12 or 24 well plates and treated for 8 hrs with fisetin (20 *μ*M), CSE (5%), chloroquine (30 *μ*M), and CFTR(inh)-172 (10 *μ*M) or transfected for 18 hrs with TFEB-Mission™ shRNA. Following this treatment, the cells were infected with *PA01-*GFP at an MOI of 10 for 3 hrs. Next, 50 or 100 *μ*l of media was collected from these cultures and spread on 2% LB agar plates supplemented with 1% carbenicillin to select for *P. aeruginosa.* These plates were incubated at 37°C for 24 hrs and bacterial colony-forming units (CFUs) were counted to quantify bacterial survival.

### 2.5. Bactericidal and Mucolytic Experiments

The 5% mucin (Sigma) solution in PBS was treated with fisetin (20 *μ*M) or cysteamine (positive control; 250 *μ*M) and stirred for 12 hrs until dissolved. The 300 *μ*l of these mucin solutions was pipetted into the top of a 1 mL sterile pipette, and the travel time to reach the bottom of the pipette was recorded. This was used to calculate velocity by dividing the length of the pipette (28 cm) by the recorded time, which was used to quantify mucolytic activity of fisetin. Finally, to evaluate the bactericidal properties of fisetin, *PA01-*GFP was cultured and treated with fisetin (20 and 40 *μ*M) or cysteamine (positive control; 250 *μ*M). The 100 *μ*l of the bacterial culture was plated in a 96-well plate and the OD (600 nm) was recorded as a measurement of bacterial growth every 3 hrs for 18 hrs.

### 2.6. Statistical Analysis

The data is presented as the mean ± standard error of the mean (SEM) and differences between the various groups were tested using standard *t*-test. The differences were considered significant if the *p* value was ≤0.05. Densitometry analysis of the results from immunoblotting and fluorescence microscopy was done utilizing ImageJ software (NIH, Bethesda, MD) as we previously described [2].

## 3. Results

### 3.1. TFEB Expression Is Induced by Fisetin in Murine Macrophages

**Figure 1:**
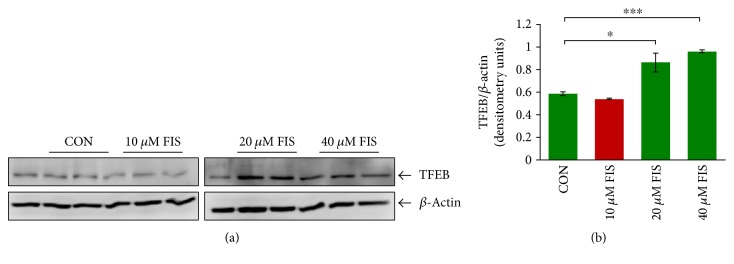
Fisetin induces TFEB expression in murine macrophages. (a) RAW264.7 cells were treated with increasing dosages of fisetin (0, 10, 20, and 40 *μ*M) for 8 hrs. Following treatment, the cells were lysed, and the total protein lysate was isolated for immunoblotting to determine changes in TFEB expression. The Western blot analysis shows an increase in TFEB (autophagy regulator; soluble) expression in the macrophages treated with 20 *μ*M and 40 *μ*M fisetin. *β*-Actin was used as a loading control. (b) Densitometry analysis of TFEB expression was normalized to *β*-actin. Data represent *n* = 4 in each group, and error bars depict mean ± SEM, ^∗^ *p* < 0.05; ^∗∗∗^ *p* < 0.001. The 20 *μ*M dose of fisetin induces a significant increase in TFEB expression, which was thus selected for further experimental investigation.

Previous publications have shown that fisetin has the ability to induce autophagy [15, 18, 19, 22]; hence, we decided to investigate its effectiveness in recovering CS-impaired phagocytosis. However, it was first necessary to determine the dose of fisetin to be used for further experimentation; thus, by treating RAW264.7 cells with increasing doses of fisetin (0, 10, 20, and 40 *μ*M) for 8 hrs, we first selected the appropriate dose. Following fisetin treatment, the total protein lysates were collected and the changes in TFEB expression were quantified. The data demonstrates that TFEB expression is significantly (*p* < 0.05 and *p* < 0.001) induced in RAW264.7 cells by both 20 and 40 *μ*M fisetin treatments (Figures 1(a) and 1(b)). Thus, 20 *μ*M fisetin dose was selected for further investigation because it was the lowest dose that induced TFEB expression.

### 3.2. CS Exposure Impairs Phagocytosis in RAW264.7 Cells

**Figure 2:**
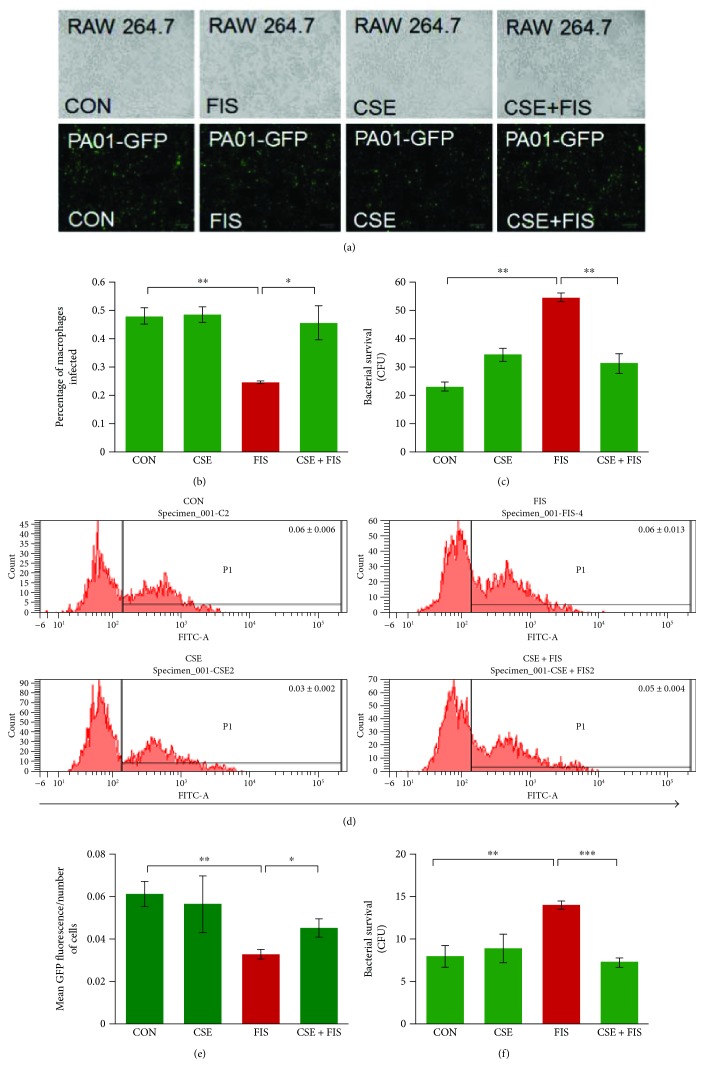
CS exposure impairs bacterial clearance by macrophages that promote bacterial survival. (a) RAW cells were pretreated with fisetin (20 *μ*M) and or CSE (5%) for 8 hrs. Following treatment, the cells were infected with *PA0*1-GFP for 3 hrs at a MOI of 10. After infection, the cells were washed twice with sterile PBS, followed by bright-field and fluorescence microscopy (scale bar, 100 *μ*m). These florescent images were utilized to quantify the number of infected cells (intracellular bacteria) using the ImageJ software. The data shows that CSE treatment significantly impairs bacterial clearance, indicated by a decrease in the number of intracellular bacteria, which was significantly recovered by fisetin. (b) The data from images shown in (a) are represented here as mean ± SEM of percentage of macrophages infected, *n* = 3, ^∗∗^ *p* < 0.01; ^∗^ *p* < 0.05. (c) The cell culture media (100 *μ*l) from the experimental groups shown in (a) were spread on 2% LB agar plates and incubated for 24 hrs at 37°C. The number of colony-forming units (CFU) was counted to quantify the number of extracellular bacteria as a representation of bacterial survival. CSE treatment resulted in significantly increased bacterial survival that was controlled by fisetin treatment. Data represents mean ± SEM of CFUs, *n* = 3, ^∗∗^ *p* < 0.01. (d) Flow cytometry results of RAW cells treated with the same groups as shown in (a) showed the CS-induced phagocytic defect as well as verify fisetin's ability to recover CSE-impaired macrophage phagocytic function. (e) The flow cytometry data expressed as mean of positive GFP florescent phagocytic cells; shown as mean ± SEM, *n* = 3, ^∗∗^ *p* < 0.01; ^∗^ *p* < 0.05. (f) Bacterial survival assay of cell culture media (100 *μ*l) from flow cytometry experiment showed a significant increase in the number of CFUs after CSE treatment, which was significantly reduced by fisetin. The data is representative of mean ± SEM of CFUs, *n* = 3, ^∗∗^ *p* < 0.01; ^∗∗∗^ *p* < 0.001. This data suggests that CS exposure impairs phagocytosis in murine macrophages that can be restored by autophagy induction.

We next investigated if CS exposure impaired phagocytosis in murine macrophages and its underlying mechanism. In these experiments, RAW cells were pretreated with fisetin (20 *μ*M) and/or CSE (5%) for 8 hrs followed by infection with PA01-GFP with an MOI of 10 for 3 hrs. Fluorescent microscopy images were captured using the Bio-Rad ZOE™ Florescent Cell Imager and analyzed using the ImageJ software. We found that CS exposure significantly (*p* < 0.01) decreased the number of intracellular bacteria as compared to controls, which was significantly (*p* < 0.01) recovered by fisetin treatment (Figures 2(a) and 2(b)). A similar trend was found with the treatment of another autophagy-inducing drug, cysteamine (see Supplementary Figure 1), which supported our mechanistic finding that CS exposure causes a phagocytic defect in murine macrophages via autophagy-impairment. To confirm this observation, the experiment was repeated and analyzed through flow cytometry. Analysis of the flow cytometry data showed that CSE-treated macrophages have a significantly (*p* < 0.01) lower number of PA01-GFP bacteria that was significantly (*p* < 0.05) increased by fisetin treatment (Figures 2(d) and 2(e)). As a functional read out, bacterial survival was quantified by plating 100 *μ*l of the cell culture media from the microscopy and flow cytometry experiments on 2% LB agar plates and incubating these plates for 24 hrs at 37°C, in order to count the colony-forming units (CFU). The plates from the CSE-treated group in both experiments had significantly (*p* < 0.01) higher bacterial survival as compared to the control, while the fisetin treatment significantly (*p* < 0.01, *p* < 0.001) diminished bacterial survival (Figures 2(c) and 2(f)). Our findings suggest that CS exposure impairs bacterial phagocytosis and increases its survival in murine macrophages that can be recovered by fisetin through autophagy induction.

### 3.3. Inhibition of Autophagy Impairs Clearance and Promotes Survival of PA01 Bacteria in Murine Macrophages

**Figure 3:**
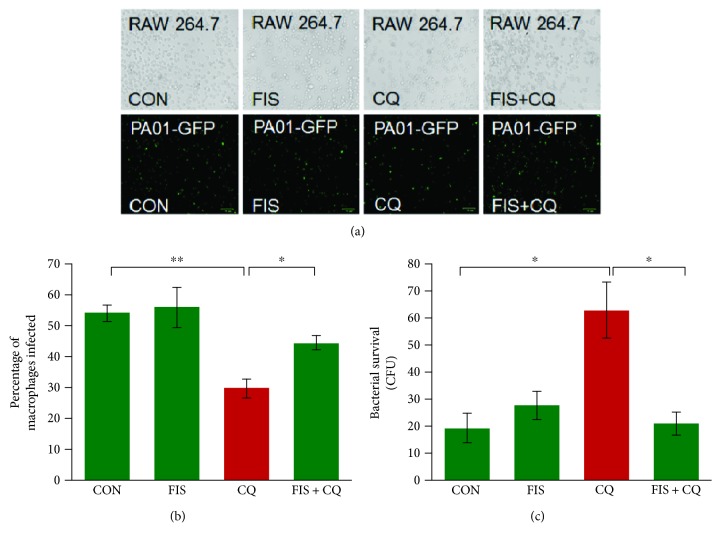
Autophagy inhibition impairs phagocytosis in murine macrophages. (a) RAW cells were pretreated with fisetin (20 *μ*M) and chloroquine (60 *μ*M) for 8 hrs. Then, the cells were infected with *PA*01-GFP for 3 hrs at a MOI of 10. After infection, the cells were washed twice with PBS followed by bright-field and fluorescence microscopy (scale bar, 70 *μ*m). ImageJ software was utilized to count the number of infected cells (intracellular bacteria). Data shows that chloroquine treatment impairs phagocytosis as indicated by the observations of significantly lower numbers of intracellular bacteria in chloroquine-treated cells, while fisetin demonstrated the ability to recover phagocytosis shown by a significant increase in the number of intracellular bacteria. (b) The data shown in (a) demonstrated the mean ± SEM of percentage of macrophages infected, *n* = 3, ^∗∗^ *p* < 0.01; ^∗^ *p* < 0.05. (c) The media (100 *μ*l) from the experimental groups in (a) were spread on 2% LB agar plates and incubated for 24 hrs at 37°C, and the number of CFUs was counted to quantify the number of extracellular bacteria as a representation of survival. Data suggests that autophagy inhibition leads to the impairment of PA01-GFP clearance, as extracellular bacterial survival was significantly higher in cells treated with chloroquine, an autophagy inhibitor. Moreover, fisetin significantly reduces the bacterial survival as anticipated. Data represents mean ± SEM of CFUs, *n* = 3, ^∗∗^ *p* < 0.01 and verifies that autophagy inhibition leads to the impairment of phagocytosis in RAW cells.

CS exposure has been found to inhibit autophagy and cause aggresome formation in chronic pulmonary diseases such as COPD [1, 14, 15, 20]. Therefore, we investigated CS-impaired autophagy as a possible mechanism for the dysfunctional phagocytosis in RAW cells (macrophages), by pretreating these cells with fisetin (20 *μ*M) and/or chloroquine (60 *μ*M) for 8 hrs. Chloroquine is an autophagy inhibitor that was utilized as a positive control for CS-impaired autophagy. Following treatment, these cells were infected with PA01-GFP at an MOI of 10 for 3 hrs as described above. Afterwards, fluorescent images were captured for analysis, which demonstrated that autophagy inhibition significantly (*p* < 0.01) impairs bacterial clearance in RAW cells that can be significantly (*p* < 0.05) recovered by fisetin treatment (Figures 3(a) and 3(b)). To further explore the effect on phagocytosis, bacterial survival was analyzed by plating the experimental media on 2% LB agar plates and performing a CFU count. The results from this bacterial survival assay showed that autophagy impairment by chloroquine significantly (*p* < 0.05) increased bacterial survival, which was significantly (*p* < 0.05) reduced by fisetin treatment (Figure 3(c)). These results verify that autophagy inhibition mediated by CS exposure results in phagocytosis dysfunction in murine macrophages.

### 3.4. TFEB Knockdown Causes a Phagocytic Dysfunction in Murine Macrophages

**Figure 4:**
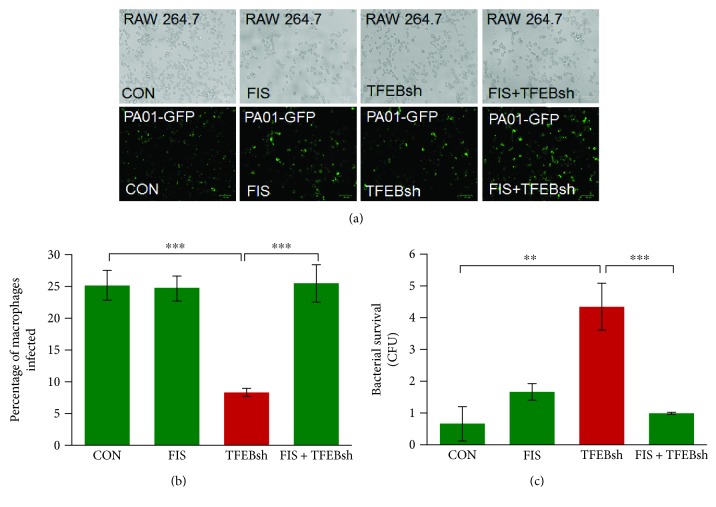
TFEB knockdown impairs phagocytosis in murine macrophages. (a) RAW cells were transfected with TFEB-Mission™ shRNA for 24 hrs. Following transfection, the cells were pretreated with fisetin (20 *μ*M) for 8 hrs. Afterwards, cells were infected with *PA0*1-GFP for 3 hrs at a MOI of 10. Following infection, the cells were washed twice with PBS followed by bright-field and fluorescence microscopy (scale bar, 70 *μ*m). The florescent images were utilized to count the number of infected cells (intracellular bacteria) using ImageJ software. The data shows that the bacterial clearance is significantly inhibited as indicated by the significantly lower number of intracellular bacteria in TFEB knockdown cells. Meanwhile, fisetin significantly restores bacterial clearance, indicated by an increase in the number of intracellular bacteria. (b) Data from A, represented as percentage of macrophages infected, which are shown as mean ± SEM, *n* = 4, ^∗∗∗^ *p* < 0.001. (c) A bacterial survival assay was performed by spreading the cell culture media (100 *μ*l) from the experimental groups in (a), on 2% LB agar plates that were incubated for 24 hrs at 37°C. CFUs were counted to quantify the extracellular bacteria survival. This data suggests that decreased TFEB expression significantly increases bacterial survival due to impaired phagocytosis. Moreover, fisetin was able to restore phagocytosis as determined by significantly fewer CFUs. The data represents mean ± SEM of CFUs, *n* = 3, ^∗∗^ *p* < 0.01; ^∗∗∗^ *p* < 0.001.

Previous research has shown that TFEB is the master autophagy regulator that initiates the transcription of various autophagy/lysosomal-related genes [15, 19, 26–28]. It has also been demonstrated that CS exposure results in TFEB localization in aggresome bodies causing decreased lung function [15]. Hence, we explored TFEB's role in autophagy- and xenophagy-impaired phagocytosis by knocking down TFEB expression in RAW cells followed by pretreatment with fisetin (20 *μ*M) for 8 hrs and infection with PA01-GFP at an MOI of 10 for 3 hrs. Fluorescent images were captured and then analyzed using the ImageJ software showing that bacterial clearance was significantly impaired (*p* < 0.001) by TFEB knockdown. Moreover, fisetin treatment was able to significantly (*p* < 0.001) rescue bacterial clearance (Figures 4(a) and 4(b)). Next, bacterial survival was quantified by counting the CFUs using cell culture media from this experiment, as described above. TFEB knockdown cells had significantly (*p* < 0.01) higher bacterial survival as compared to that of the control that was controlled significantly (*p* < 0.001) by treatment with fisetin (Figure 4(c)). Therefore, the data suggests that the regulation of autophagy by TFEB has a role in the CS-impaired phagocytosis.

### 3.5. CFTR Inhibition Impairs Phagocytosis in RAW264.7 Cells

**Figure 5:**
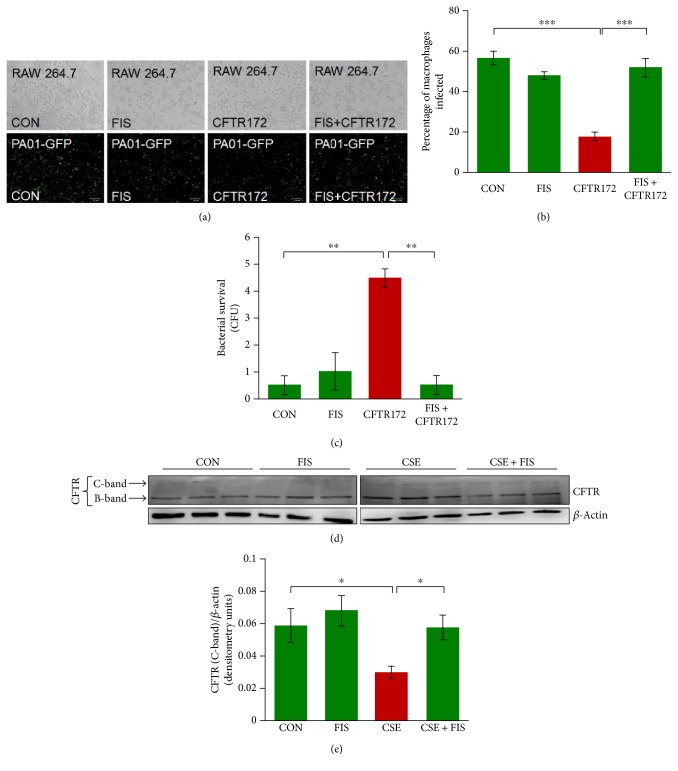
Fisetin recovers phagocytosis defect mediated by CFTR inhibition. (a) RAW cells were pretreated with CFTR172 inhibitor and/or fisetin (20 *μ*M) for 8 hrs followed by infection with *PA01-GFP* for 3 hrs at a MOI of 10. After infection, the cells were washed twice with PBS and observed using bright-field and fluorescence microscopy (scale bar, 70 *μ*m). The fluorescent images were used to count the number of macrophages infected (intracellular bacteria) using the ImageJ software. The data shows that the inhibition of CFTR impairs phagocytosis shown by a significantly lower number of intracellular bacteria, which was significantly recovered by fisetin treatment. (b) The data from (a) represented as the mean ± SEM of percentage of macrophages infected, *n* = 3, ^∗∗∗^ *p* < 0.001. (c) A bacterial survival assay was performed using the cellular media (100 *μ*l) from experimental groups in A, which were plated on 2% LB agar plates and incubated for 24 hrs at 37°C. The colony-forming unit bacterial counts show that CFTR inhibition results in significantly higher numbers of CFUs representing impaired phagocytosis, which was significantly recovered by fisetin treatment. The data represents mean ± SEM of CFUs, *n* = 2, ^∗∗^ *p* < 0.01. (d) RAW cells were treated with CSE and/or fisetin (20 *μ*M) for 8 hrs. After treatment, the total protein lysate was isolated and analyzed by immunoblotting for changes in the expression of CFTR and p62 expression. The Western blot analysis shows that membrane CFTR expression (C-band) was significantly decreased by CSE exposure, which was significantly recovered upon fisetin treatment. *β*-Actin was used as a loading control. (e) Densitometry analysis of CFTR and p62 expression was normalized to *β*-actin. Data represent *n* = 3 in each group, and error bars depict mean ± SEM, ^∗∗^ *p* < 0.05. Thus, data shows that CFTR inhibition affects the phagocytic ability of macrophages. Moreover, fisetin shows the ability to correct this CFTR-mediated phagocytic defect in RAW cells.

CS exposure has been demonstrated to cause CFTR dysfunction through accumulation in aggresome bodies [2, 25, 29, 30]. This dysfunction has been associated with impaired autophagy and increased bacterial colonization [2, 31–34]. Thus, we investigated the role of CFTR dysfunction in impaired phagocytosis by pretreating RAW cells with the CFTR172 inhibitor (10 *μ*M) and/or fisetin (20 *μ*M) for 8 hrs followed by PA01-GFP infection with an MOI of 10 for 3 hrs. The analysis of fluorescent images showed that inhibition of CFTR significantly (*p* < 0.001) impaired bacterial clearance compared to the control, which was significantly (*p* < 0.001) recovered by fisetin treatment (Figures 5(a) and 5(b)). The phagocytic function of RAW cells was further evaluated by plating the cell culture media from this experiment on 2% LB agar plates for 24 hrs at 37°C and counting the CFUs in order to quantify bacterial survival. These results demonstrated that CFTR inhibition significantly (*p* < 0.01) increased bacterial survival, which was significantly (*p* < 0.01) decreased by fisetin treatment (Figure 5(c)). These results suggest that CFTR dysfunction also has a role in phagocytosis impairment. In order to investigate if fisetin can recover the CS-induced CFTR dysfunction, RAW cells were treated with CSE (5%) and/or fisetin (20 *μ*M) for 8 hrs, and the total protein lysates were isolated. Immunoblotting was utilized to quantify changes in CFTR that demonstrates significant (*p* < 0.05) decrease in membrane CFTR expression (C-band) with CSE exposure, which was significantly (*p* < 0.05) recovered by fisetin treatment (Figures 5(d) and 5(e)). Thus, the data suggests that CSE decreases membrane CFTR expression in murine macrophages, potentially due to CFTR misfolding, CS-impaired autophagy, and CFTR aggresome accumulation, further aggravating phagocytic response.

### 3.6. Fisetin Shows Promise as Bactericidal and Mucolytic for Treatment of COPD

**Figure 6:**
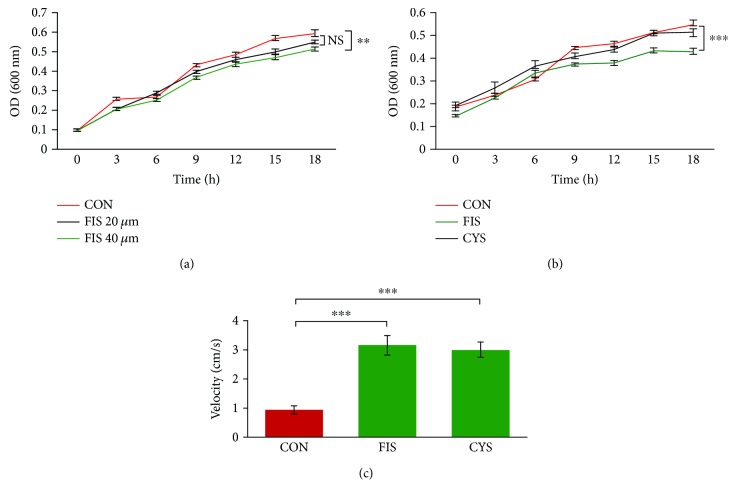
Fisetin demonstrates bactericidal and mucolytic properties. (a) *PA01* was grown for 15 hrs in LB broth then treated with fisetin (20 and 40 *μ*M). Upon treatment, the OD (600 nm) was recorded to quantify the number of bacteria in the culture. Subsequently, the OD was taken every 3 hrs for 18 hrs to analyze changes in bacteria proliferation. The 40 *μ*M fisetin demonstrates a significant inhibition of bacterial growth compared to the control. The data represents mean ± SEM, *n* = 3, ^∗∗^ *p* < 0.01. (b) The experimental procedure from (a) was repeated with fisetin (40 *μ*M) or cysteamine (250 *μ*M). The data shows that fisetin significantly reduces the number of bacteria compared to the control and cysteamine treatment. Data represents mean ± SEM, *n* = 5, ^∗∗∗^ *p* < 0.001. (c) The 5% mucin solution was stirred overnight with or without fisetin (20 *μ*M) or cysteamine (250 *μ*M). The 300 *μ*l of this mucin mix was then pipetted into the top of a 1 mL sterile pipette, and the velocity of the mucin was recorded as a representation of changes in mucus viscosity or mucolytic activity. The data shows that both cysteamine (positive control) and fisetin significantly decreased the viscosity of mucin suggesting fisetin has a mucolytic potential. Data represents mean ± SEM, *n* = 5, ^∗∗∗^ *p* < 0.001.

The pathogenesis of many chronic pulmonary diseases involves chronic inflammation, recurrent exacerbations, and mucus buildup [2, 13, 17, 31, 35–39]. Thus, a therapeutic approach that controls these disease characteristics would be ideal for dealing with chronic exacerbations. Cysteamine is one such drug that has known antioxidant, anti-inflammatory, autophagy-inducing, mucolytic, and bactericidal properties [13, 14, 40, 41]. Similarly, fisetin (over-the-counter medication) also has antioxidant and anti-inflammatory properties, along with being a potent autophagy-inducing drug [15, 23, 42–44]. Furthermore, recent studies have shown that effective killing of various bacteria and viruses [45–48] can help with chronic or recurrent exacerbations. Hence, we investigated the bactericidal properties of fisetin against PA01-GFP. To do so, PA01 was grown for 15 hrs in LB broth followed by treatment with fisetin (20 or 40 *μ*M). Upon treatment, the OD (600 nm) was recorded to quantify the number of bacteria in the culture. This was repeated every 3 hrs for 18 hrs. Analysis of the changes in OD showed that 40 *μ*M fisetin significantly (*p* < 0.01) inhibited bacterial proliferation. Next, this experiment was repeated with fisetin (40 *μ*M) or cysteamine (250 *μ*M) treatment. This data verified that 40 *μ*M fisetin treatment significantly (*p* < 0.001) impaired bacterial growth suggesting its bactericidal properties (Figures 6(a) and 6(b)). Next, we focused on the mucolytic potential of fisetin by stirring 5% mucin solutions overnight with or without fisetin (20 *μ*M) or cysteamine (positive control; 250 *μ*M). The mucin mix (300 *μ*l) was then pipetted into the top of a 1 ml sterile pipette, and the velocity of mucin was recorded as a representation of changes in mucus viscosity. The data showed that both fisetin and cysteamine treatment significantly (*p* < 0.001) decreased the viscosity of the mucin solution suggesting that fisetin has mucolytic potential (Figure 6(c)). These findings suggest that fisetin may be an effective treatment of recurrent or chronic exacerbations in COPD subjects.

## 4. Discussion

**Figure 7:**
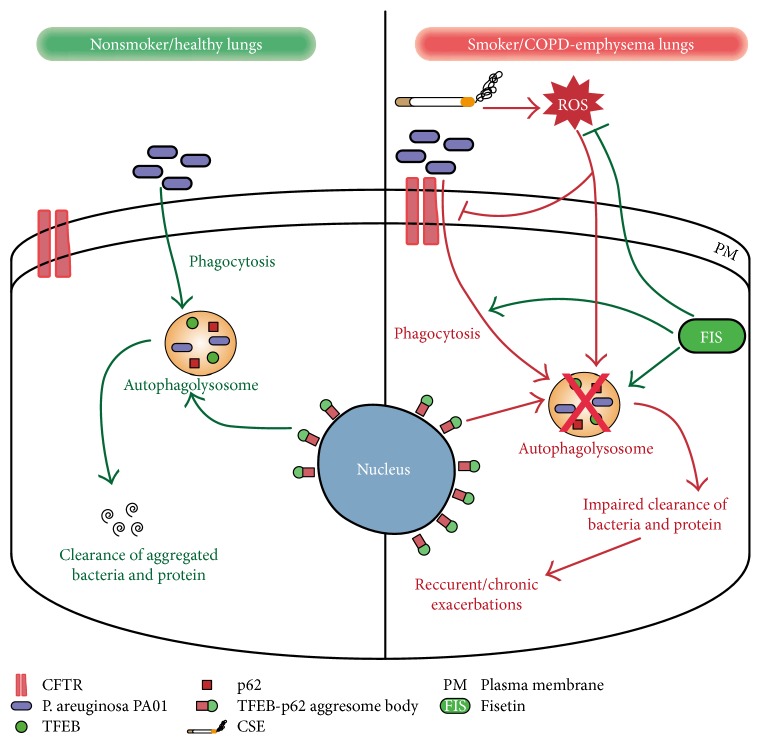
Schematic representation showing the mechanism of cigarette smoke-induced exacerbations in COPD via TFEB-mediated autophagy impairment. Our mechanistic analysis shows that CS/ROS exposure causes TFEB accumulation in aggresome bodies, thus impairing expression of autophagy-regulating proteins leading to chronic autophagy inhibition. Furthermore, CS exposure also causes a decrease in CFTR membrane expression, due to its similar accumulation in aggresome bodies further aggravating autophagy. This CS-mediated chronic autophagy impairment induces a phagocytic defect that results in an increase in bacterial infection as a mechanism for recurring or chronic exacerbations in COPD-emphysema subjects. Moreover, treatment with over-the-counter antioxidant medication, fisetin that induces TFEB expression to restore levels of autophagy proteins, corrects the CS-induced phagocytic defect. Thus, suggesting therapeutic potential of fisetin or autophagy-inducing drugs in controlling recurrent exacerbations in COPD-emphysema subjects.

One aspect of CS-induced COPD-emphysema pathogenesis is constant bacterial colonization of the lower airways that provoke recurrent exacerbations in subjects causing high morbidity and mortality [2, 49–51]. Studies have suggested that CS exposure impairs phagocytosis, which may account for these exacerbations [2, 11, 52–54]; however, the mechanism was unknown. Our studies first verified that CS exposure significantly impaired bacterial (*P. aeruginosa* PA01-GFP) phagocytosis and improved its survival in murine macrophages. Next, to investigate a possible underlying mechanism for this dysfunction, we focused on autophagy and xenophagy, which is impaired by CS exposure. As a model of CS-induced autophagy and xenophagy impairment, we treated RAW cells with chloroquine, an autophagy inhibitor (as a positive control), and found that bacterial clearance was significantly hindered and its survival was significantly increased. Since, CS-impaired autophagy showed promise as an underlying mechanism for the phagocytic defect found in COPD-emphysema that can lead to recurrent exacerbations, we further investigated this process. We observed that the knockdown of master autophagy regulator, TFEB expression, in RAW cells significantly impaired the clearance of PA01-GFP and significantly enhanced its survival. This finding is important in understanding the mechanism because TFEB is the master autophagy regulator [15, 19]; thus, decreasing its expression leads to impairment of autophagy and xenophagy that we found to be a key regulator of phagocytosis in the murine macrophages. Hence, we concluded that CS-induced autophagy impairment might account for dysfunctional phagocytosis in COPD-emphysema subjects, the mechanism for which is described in Figure 7. Moreover, previous investigations have demonstrated the role of CFTR in the regulation of phagocytosis [2, 25, 33], as well as acquired CFTR dysfunction induced by CS exposure. Consequently, we found that inducing CFTR dysfunction with CFTR inhibitor 172 significantly impaired bacterial phagocytosis and increased its survival in RAW cells, which suggest CFTR has a role in CS-induced phagocytosis that regulates TFEB-mediated autophagy. Therefore, our investigation shows for the first time that CS-impaired autophagy and xenophagy functions mediate an acquired CFTR defect as the underlying mechanism for the phagocytic defect found in COPD.

Moreover, it has been shown that CSE is high in reactive oxidant species (ROS) which has a deleterious effect on many cellular processes and respiratory function. Furthermore, ROS and CSE have been shown to inhibit autophagy [18, 20, 55], which supports our findings that CS-impaired autophagy has a role in the phagocytosis dysfunction observed in macrophages. Furthermore, ROS causes CFTR dysfunction [2, 25, 56], which we found to further exacerbate CS-induced autophagy impairment resulting in phagocytic defect. Thus, CS-induced autophagy and xenophagy impairment and CFTR dysfunction are synergistic mechanisms mediating phagocytic dysfunction seen in COPD subjects. Hence, we investigated if this phagocytosis dysfunction could be alleviated through the use of an autophagy mediating, antioxidant drug, fisetin. Fisetin is a flavonoid that has anti-inflammatory and antitumorigenic properties [15, 23, 44, 57]. Fisetin has also been shown to function as an autophagy inducer [15]. In this study, we found that induction of autophagy by an antioxidant, fisetin, significantly restored the CS-impaired phagocytic function and thus significantly reduced bacterial survival in RAW cells. Fisetin was also able to significantly restore phagocytosis when CFTR function was inhibited. These findings suggest that fisetin or other autophagy-inducing antioxidant drugs offer a novel therapeutic approach for the treatment of recurrent exacerbations in COPD-emphysema by targeting the mechanistic role of autophagy and xenophagy in phagocytosis dysfunction. This is significant because the constant treatment of these exacerbations with antibiotics leads to resistance in bacteria, resulting in increased mortality [49, 58, 59]. Thus, by focusing on restoring the phagocytic function of macrophages in COPD subjects instead of targeting the bacteria directly, it is possible to prevent recurrent exacerbations and bacterial resistance in patients with COPD.

Furthermore, COPD and other chronic pulmonary diseases are characterized by inflammation, oxidative stress, increased mucus secretion, and bacterial infection [7, 12, 35, 38, 39, 60–62]. Hence, ideal treatments for these diseases would address all of these aspects instead of a treatment targeting only one specific characteristic of chronic obstructive pulmonary disease(s). Cysteamine is one such drug with properties that have been shown to influence these characteristics of chronic respiratory disease pathogenesis [13, 14, 41]; therefore, we investigated fisetin's therapeutic abilities in comparison to cysteamine (as a positive control). Fisetin has known antioxidant, anti-inflammatory, and bactericidal properties against some bacteria [23, 57]. Moreover, our investigation showed that fisetin could act as a mucolytic with comparable effectiveness to cysteamine, as well as inhibit the growth of PA01-GFP. These findings suggest that fisetin has promise in treating other pathogenic characteristics of obstructive lung diseases such as COPD-emphysema and cystic fibrosis. However, further investigation is necessary into the exact chemical properties of fisetin that account for its mucolytic and direct bactericidal activity.

Despite our findings of direct bactericidal activity in fisetin, this property only accounted for a 1.2-fold decrease in bacterial survival in our experiments. Meanwhile, in our phagocytosis experiments, the decrease in bacterial survival was fourfold; thus, it can be asserted that the significant decrease in bacterial survival seen in the fisetin-treated groups in these experiments was a result of autophagy induction. This assertion is possible because the effect of fisetin's direct bactericidal affect was minimal in comparison to our observations in phagocytosis-mediated bacterial clearance, which meant the restoration of phagocytosis-influenced bacterial killing in these results. This further supports that CS impairs TFEB-mediated autophagy as a mechanism for the phagocytic defect in COPD-emphysema subjects, as well as fisetin's therapeutic potential to restore phagocytosis through TFEB induction. Moreover, CS-acquired CFTR dysfunction is amplified by TFEB-mediated autophagy impairment as this results in aggresome arrest of CFTR as we recently described [25, 56]. Thus, as anticipated, autophagy-inducing antioxidants such as fisetin and cysteamine not only augment CS-impaired autophagy but also the resulting CFTR dysfunction and phagocytic defect.

## 5. Conclusion

In conclusion, we establish CS-impaired autophagy and xenophagy as a critical mechanism involved in the resulting phagocytic defect. Furthermore, autophagy-inducing drugs with anti-oxidant characteristics such as fisetin restore CS-impaired phagocytosis demonstrating its therapeutic potential in controlling recurrent exacerbations in COPD-emphysema and other chronic respiratory diseases.

## Supplementary Materials

Supplementary materials Supplementary Figure 1. Cysteamine recovers CS induced phagocytic defect in murine macrophages. (A) The Raw cells were pre-treated with cysteamine (250 μM) and/or CSE (5%) for 8hrs. After treatment, the cells were infected with *PA0*1-GFP for 3hrs at a MOI of 10. Then, the cells were washed twice with sterile PBS, followed by bright field and fluorescence microscopy (scale bar, 70 μm). These florescent images were utilized to quantify the number of infected cells (intracellular bacteria) using the ImageJ software. The data shows that CSE treatment significantly impairs bacterial clearance, indicated by a decrease in the number of intracellular bacteria, which was significantly recovered by cysteamine treatment. (B) The data from images shown in A are represented here as mean ± SEM of percentage of macrophages infected, *n* = 3, ^∗∗∗^ *p* < 0.001. (C) The cell culture media (100 μl) from the experimental groups shown in A were spread on 2% LB-agar plates and incubated for 24 hrs at 37°C. The number of colony forming units (CFU) was counted to quantify the number of extracellular bacteria as a representation of bacterial survival. CSE treatment resulted in significantly increased bacterial survival. Treatment with cysteamine showed a decrease in bacterial survival; however, it was insignificant. Data represents mean ± SEM of CFUs, *n* = 3, ^∗^ *p* < 0.05.
