# Supplementary material for: Cigarette Smoke Exposure Inhibits Bacterial Killing via TFEB-Mediated Autophagy Impairment and Resulting Phagocytosis Defect
Source: Mediators Inflamm. 2017 Dec 28;2017:3028082. doi: 10.1155/2017/3028082 (PMC5763241; doi:10.1155/2017/3028082)

Supplementary Figure Legend

Supplementary Figure 1. Cysteamine recovers CS induced phagocytic defect in murine macrophages. (A) The Raw cells were pre-treated with cysteamine (250 µM) and/or CSE (5%) for 8hrs. After treatment, the cells were infected with *PA0*1-GFP for 3hrs at a MOI of 10. Then, the cells were washed twice with sterile PBS, followed by bright field and fluorescence microscopy (scale bar, 70 µm). These florescent images were utilized to quantify the number of infected cells (intracellular bacteria) using the ImageJ software. The data shows that CSE treatment significantly impairs bacterial clearance, indicated by a decrease in the number of intracellular bacteria, which was significantly recovered by cysteamine treatment. (B) The data from images shown in A are represented here as mean ± SEM of percentage of macrophages infected, n=3, ***$p < 0.001$. (C) The cell culture media (100 µl) from the experimental groups shown in A were spread on 2% LB-agar plates and incubated for 24hrs at 37ºC. The number of colony forming units (CFU) was counted to quantify the number of extracellular bacteria as a representation of bacterial survival. CSE treatment resulted in significantly increased bacterial survival. Treatment with cysteamine showed a decrease in bacterial survival; however, it was insignificant. Data represents mean ± SEM of CFUs, n=3, *$p < 0.05$.

Supplementary Fig. 1

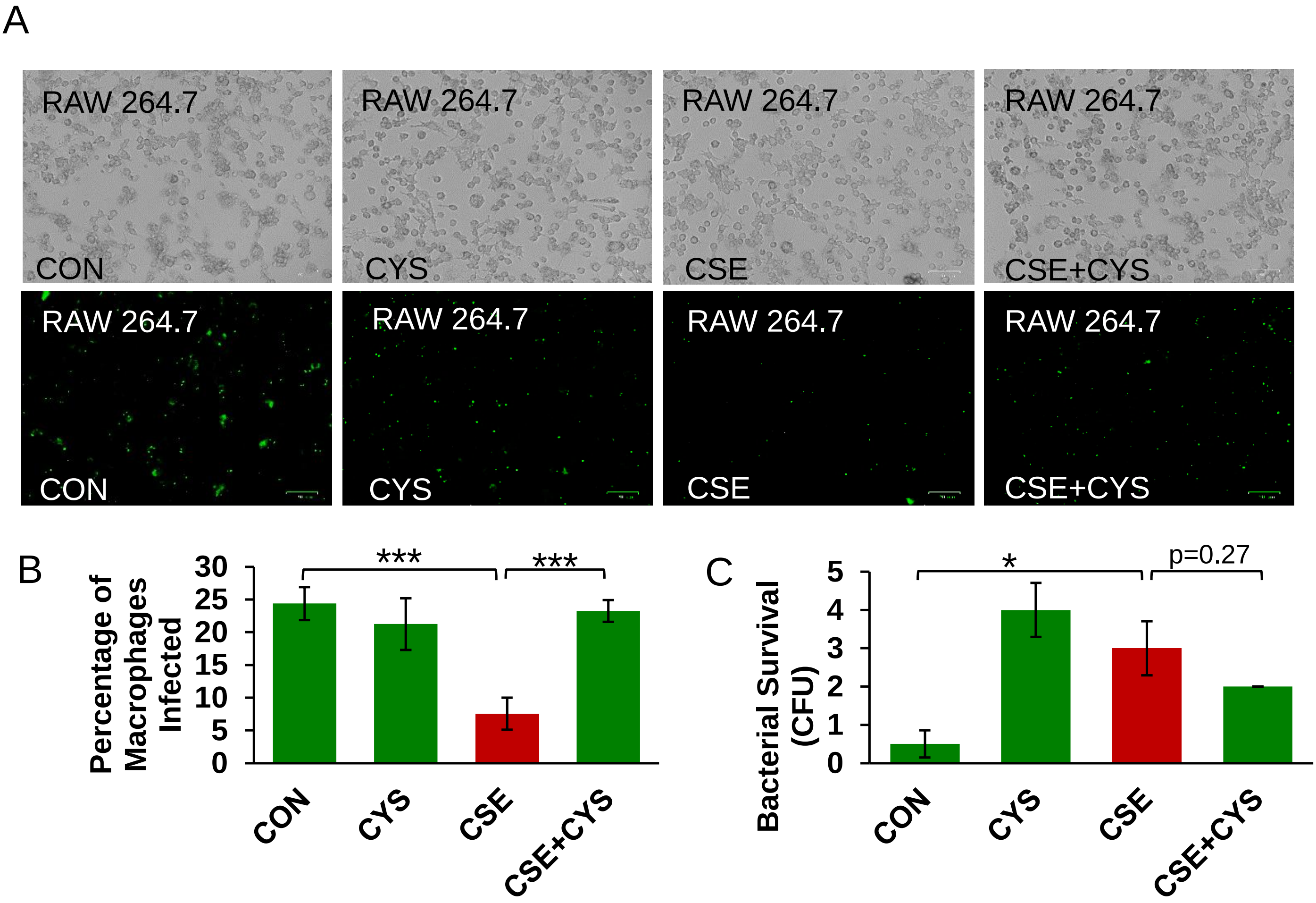

Supplement: Supplementary materials — Supplementary Figure 1. Cysteamine recovers CS induced phagocytic defect in murine macrophages. (A) The Raw cells were pre-treated with cysteamine (250 μM) and/or CSE (5%) for 8hrs. After treatment, the cells were infected with PA01-GFP for 3hrs at a MOI of 10. Then, the cells were washed twice with sterile PBS, followed by bright field and fluorescence microscopy (scale bar, 70 μm). These florescent images were utilized to quantify the number of infected cells (intracellular bacteria) using the ImageJ software. The data shows that CSE treatment significantly impairs bacterial clearance, indicated by a decrease in the number of intracellular bacteria, which was significantly recovered by cysteamine treatment. (B) The data from images shown in A are represented here as mean ± SEM of percentage of macrophages infected, n = 3, ∗∗∗ p < 0.001. (C) The cell culture media (100 μl) from the experimental groups shown in A were spread on 2% LB-agar plates and incubated for 24 hrs at 37°C. The number of colony forming units (CFU) was counted to quantify the number of extracellular bacteria as a representation of bacterial survival. CSE treatment resulted in significantly increased bacterial survival. Treatment with cysteamine showed a decrease in bacterial survival; however, it was insignificant. Data represents mean ± SEM of CFUs, n = 3, ∗ p < 0.05. [file 3028082.f1.pdf]
